# Segmentation Methodologies for the Construction of Hyperspectral Cell Nuclei Databases in Histopathology

**DOI:** 10.3390/bioengineering13030306

**Published:** 2026-03-05

**Authors:** Gonzalo Rosa-Olmeda, Sara Hiller-Vallina, Manuel Villa, Berta Segura-Collar, Ricardo Gargini, Miguel Chavarrías

**Affiliations:** 1 CEIMM, Center for Industrial Electronics & Multimodal Systems, Universidad Politécnica de Madrid, 28031 Madrid, Spain; gonzalo.rosa.olmeda@upm.es (G.R.-O.); manuel.villa.romero@upm.es (M.V.); 2 Instituto de Investigación Biomédicas I+12, Hospital Universitario 12 de Octubre, 28041 Madrid, Spain; 3 Pathology and Neurooncology Unit, Hospital Universitario 12 de Octubre, 28041 Madrid, Spain

**Keywords:** histopathology, hyperspectral, nuclei segmentation, microscopy, deep learning, computer vision, biomedicine, tumor

## Abstract

Hyperspectral imaging (HSI) extends conventional histopathology by combining spatial morphology with rich spectral information that reflects tissue biochemical composition, offering new opportunities for quantitative tissue analysis. However, reliable spectral analysis requires accurate instance-level segmentation of cell nuclei to enable the construction of meaningful nuclear spectral databases. In this work, a comprehensive methodology for generating hyperspectral databases of cell nuclei from histopathological samples is presented, including hyperspectral acquisition, preprocessing, nucleus segmentation, and spectral signature extraction. Three nucleus segmentation methods are evaluated: a spectral-only approach based on pixel-wise hyperspectral signatures in the visible–VNIR range; a spatial-only approach using synthetic RGB images derived from hyperspectral cubes; and a combined spatial–spectral approach that jointly exploits spatial and spectral information. The methods are assessed on a proprietary dataset of 30 hyperspectral cubes of tumor and healthy histopathological brain tissue annotated by expert pathologists. The spectral-only method achieves a Dice similarity coefficient (DSC) of 61.89% and produces severe over-segmentation, with cell count deviations exceeding substantially the ground truth in healthy tissue. The spatial-only method attains the highest pixel-wise accuracy (78.97% DSC) but underestimates nucleus counts by approximately 30% in tumor regions due to nucleus merging. The spatial–spectral method achieves a DSC of 73.13% and a mean cell count deviation of 4%, providing more reliable instance-level separation. These findings demonstrate that pixel-wise accuracy alone is insufficient for hyperspectral nuclear database generation.

## 1. Introduction

In recent years, unprecedented advances in medical imaging have significantly transformed cancer diagnosis. Techniques such as magnetic resonance imaging (MRI), computed tomography (CT), and positron emission tomography (PET) provide detailed spatial and functional information, making them crucial for the detection, characterization, and monitoring of tumors, as well as a guide for therapeutic planning [1]. Advances in diagnostic technologies require that image processing not be limited to isolated technical solutions, rather that it be integrated into them. In this context, multimodal and multiomic integration is increasingly recognized as a core paradigm in modern biomedical research [2], such as the integration of pathology and radiology images with genomic and clinical data [3], as it enables a more comprehensive characterization of complex phenotypes by integrating complementary signals across different scales (the molecular and cellular, and at the tissue level). Moreover, this transition to quantitative biomedical imaging enables us to leverage the ability of deep learning models to extract morphological details, textures, and complex patterns that often exceed the visual perception of human experts, enabling more objective clinical decision-making [4,5].

Currently, histopathology remains the gold standard for tumor diagnosis. Pathologists can identify and categorize neoplastic lesions with high specificity by looking at cell morphology and tissue architecture [6,7]. However, histopathological assessment is still largely manual and time-consuming, as well as being heavily dependent on the training and expertise of each pathologist. In addition, it requires a series of molecular markers that are quite time-consuming and significantly increase the cost of diagnosis and routine pathology services. This complicates efforts to scale or standardize diagnostics, especially in high-volume or resource-limited settings. In response to these challenges, deep learning (DL)-based tools have been applied for assisted image diagnosis, enabling faster and more reproducible analysis of whole-slide image (WSI) captures [8] while maintaining high diagnostic performance, as shown in recent GBM studies [9,10].

Although all these advances in DL-based pathological diagnostic tools are crucial, they are all based on information from standard red, green, and blue (RGB) cameras, which limits their ability to capture biochemical tissue properties beyond what the professional eye can see. In this context, histopathology can benefit substantially from the application of hyperspectral imaging (HSI). This technology preserves one of the fundamental pillars of histopathology, which is the morphological analysis of tissues through spatial information, while simultaneously enabling the investigation of the tissue’s other spectral information. Moreover, optical microscopy environments provide nearly ideal acquisition conditions for hyperspectral imaging due to controlled illumination and minimal external light interference. The data acquired by this type of imagery are called hyperspectral cubes, and consist of spatial images with an additional spectral dimension spanning multiple contiguous wavelength bands. Building upon these technical capabilities, HSI has gained increasing attention. Variations in spectral signatures can reveal subtle biochemical and metabolic alterations reflecting changes in the tissue [11]. An alteration in tissue that leads to cancer often affects the morphology of tumor cells depending on proliferation, tumor microenvironment resources, and specific metabolic dispositions [12]. For instance, HSI has been applied to quantitatively measure oxygenation levels in tissues being tested for various pathologies [13]. These findings highlight the ability of HSI to detect subtle biochemical changes beyond morphological changes at the cellular level, reinforcing its potential as a powerful diagnostic tool.

Indeed, hyperspectral imaging has been increasingly explored in biomedical applications. For instance, Karim et al. [14] provide a comprehensive review of medical fields in which HSI is currently being applied, while Mangotra et al. [15] present an extensive compilation of preprocessing techniques, evaluation metrics, and clinical applications of HSI for early disease diagnosis. Within the context of conventional histopathology based on hematoxylin and eosin (H&E) staining, recent studies have demonstrated the feasibility and reliability of HSI for histological analysis. Yan et al. [16], for example, highlight the applicability of HSI to lung cancer specimens and propose labeling strategies aimed at reducing the annotation burden of pathologists. Jong et al. [17] investigate hyperspectral analysis of surgical margins in breast cancer resections using H&E-stained samples and spectral unmixing techniques. Alternative hyperspectral modalities have also been explored, such as in the work by Muniz et al. [18], which analyzes healthy and tumor tissues using Fourier transform infrared hyperspectral imaging (FTIR-HSI). In this context, hyperspectral microscopic imaging (HMI) is one of the most promising tools assisting pathologists in the accurate detection of abnormalities in the tissues analyzed.

The aim of this paper is to establish a reliable, repeatable, and effective methodology that allows for the generation of hyperspectral databases of cell nuclei regardless of the tissue analyzed, in order to enable subsequent analyses based on spectral properties. In histopathology, the cell nucleus plays a central role in disease diagnosis and grading as nuclear morphology reflects fundamental biological processes such as chromatin organization, cell cycle progression, proliferation, and genetic instability. Indeed, many diagnostic criteria routinely used by pathologists, such as nuclear size, shape, density, pleomorphism, and chromatin texture, are primarily derived from nuclear features rather than from cytoplasmic or stromal components. In order to extract spectral information solely from cell nuclei, we have proposed three different segmentation methodologies that were compared by evaluating both pixel-wise segmentation accuracy and instance-level nucleus separation. We have also analyzed the practical implications of each method for downstream spectral analysis.

To the best of the authors’ knowledge, the systematic evaluation of nuclei segmentation strategies for hyperspectral histopathological images, specifically in the context of reliable nuclear spectral database generation, has not yet been thoroughly explored. Existing works have addressed related problems from different perspectives but fall short of this specific goal. For instance, Sultana et al. [19] propose a U-Net-based pipeline for cytoplasm segmentation, emphasizing hyperspectral-specific data augmentation strategies that simulate instrumental noise from raw CMOS sensor data to improve robustness. Ma et al. [20] introduce a patch-based classification framework in which hyperspectral cubes are reduced using principal component analysis (PCA) and subsequently classified using neural networks, demonstrating the relevance of hyperspectral information for tissue diagnosis; however, nuclear regions are not explicitly segmented, limiting the correct extraction of nucleus-specific spectral signatures. Chen et al. [21] present a segmentation approach based on binarization of a specific spectral band exploiting chromatin distribution, followed by cell detection using support vector machines (SVMs), but this method is highly dependent on acquisition conditions and staining consistency, restricting its generalizability. Finally, Yun et al. [22] propose SpecTr, a U-Net-based segmentation framework that employs transformers to filter redundant or noisy spectral bands; nevertheless, its reported segmentation performance remains limited. Collectively, these studies highlight the growing interest in hyperspectral histopathology while underscoring the lack of a comprehensive and comparative analysis focused on robust nucleus segmentation for hyperspectral signature extraction.

Therefore, the main contributions of this research work are as follows:

- The development of a complete methodology for the generation and evaluation of hyperspectral databases of cell nuclei from histopathological samples.
- The proposal and evaluation of three nucleus segmentation strategies: a purely spectral approach, a purely spatial approach, and a combined spatial–spectral approach.
- A systematic comparison of the proposed methods using pixel-wise segmentation metrics, instance-level cell count analysis against ground truth, and qualitative evaluation of the resulting segmentation maps.
- The identification of an optimal pipeline for hyperspectral nucleus database generation based on a 3D U-Net architecture, achieving a Dice similarity coefficient (DSC) of 73.13% for nucleus segmentation and a cell count deviation of approximately 4% relative to ground truth, indicating robust instance-level nucleus separation.

## 2. Materials and Methods

In order to study how different segmentation strategies impact the generation of hyperspectral databases of cell nuclei, we designed and evaluated three segmentation methodologies representing distinct modeling paradigms. These approaches uses learning-based methods exploiting spectral information, spatial context, or their joint spatial–spectral representation. All methods aim to produce binary nucleus masks from hyperspectral image cubes, which are subsequently used to extract nuclear spectral signatures. By comparing these methodologies under a common experimental protocol, we seek to identify which type of information plays a dominant role in accurate nucleus delineation and, consequently, in the quality of the resulting hyperspectral database.

### 2.1. Dataset

#### 2.1.1. Patient Cohort

The histopathological samples used to generate the dataset used in this article belong to the cohort of brain tissue patients presented in Section 2.1 of our previous work [9]. This cohort of patients belongs to the Hospital Universitario 12 de Octubre de Madrid (Madrid, Spain) where the samples were collected following the patient’s written informed consent and with the approval of the Ethics Committee of the hospital (CEI: 21/551 and 24/084). The brain tissue samples were stained with hematoxylin and eosin following the staining process described in Section 2.2 of our previous work mentioned above. To generate the dataset used in this study, expert pathologists from the Hospital Universitario 12 de Octubre selected 10 highly representative brain tumor tissue samples and 20 highly representative healthy brain tissue samples from patients without any history of cerebral damage or neurodegenerative disease. In this way, we obtained a dataset representing brain tissue with great heterogeneity.

#### 2.1.2. Hyperspectral Image Acquisition

Once the samples were selected, they needed to be captured as hyperspectral image. The system used for capturing the dataset in the presented work is composed by an Olympus BX-51 optical brightfield microscope (Olympus, Tokyo, Japan) equipped with a motorized stage X-Y Prior H101BXDK (Prior Scientific Instruments, Fulbourn, Cambridge, UK) and a 100W halogen transmittance light source (Phillips, Amsterdam, The Netherlands). This is a multimodal image capture system, as it captures RGB images and hyperspectral images. The RGB camera is a Basler ace acA5472-17uc RGB camera (Basler, Ahrensburg, Germany) and the hyperspectral camera is a wedge line scan Ximea MQ022HG-IM-LS150-NIR (Ximea, Münster, Germany) with a raw resolution of 2048 × 1088, a pixel size of 5.5 µm, a bit depth of 8 bits and a spectral range from 470 to 890 nm in 145 spectral bands. The HS camera used in this work is based on line scan technology, which requires a relative motion between the camera and the sample. In the setup, this motion is provided by the motorized stage. The firmware responsible for controlling the motorized stage and synchronizing its movement with frame acquisition was entirely developed by the authors [23]. In the mentioned publication, details of the methodology for capturing the samples are provided. However, for the reader’s convenience, a brief summary of this methodology is provided here.

- Starting with a prepared slide with a histopathological sample stained with H&E, the sample is digitized by capturing it as a whole-slide image using a magnification of 4× with the RGB camera.
- These WSIs are analyzed in a proprietary labeling tool software [23] where pathologists mark which areas of interest will be captured subsequently. To do this, the tissue regions are marked with fixed-size bounding boxes that are assigned a pathology label that may correspond to tumoral and non-tumoral areas, healthy tissue, vessels, necrotic zones, peripheral regions, or edematous areas.
- The coordinates of these regions of interest are then processed by the microscope’s firmware, which re-acquires the corresponding areas at a 10× magnification This automatic sequence ensures that all images taken are completely in-focus and under the same exposure time, thus ensuring standardization of the dataset. In addition, this last step compresses the HS raw frames into an .mp4 video file using an H265 [24] video compressor with a lossless ratio of 1:8:1. The decision to use a 10× magnification was made to capture the widest possible microenvironment for each sample, providing an effective balance between spatial detail and the amount of information contained in each capture.

Therefore, this methodology generates as many hyperspectral cubes for each sample as there are areas of interest marked by the pathologist during their evaluation of the sample.

#### 2.1.3. Hyperspectral Imaging Preprocess

After the sequence of automatic scans described in the previous section, the raw frames are acquired. These frames need to be preprocessed to be able to analyze the spectral information in them. To do so, we propose a six-step preprocessing chain, as outlined below.

**Step 1: Image calibration**

After decompressing the raw frames, each frame is calibrated to to homogenize hyperspectral information captured under the same lighting conditions by using white and black reference images. The white reference image is captured on the same glass as the samples, without tissue and under the same lighting conditions and camera settings, and the black reference image is captured with the same camera settings but with the optical channel completely covered so that only the camera’s baseline noise (dark current) is captured. For each frame, the calibration equation, Equation (1), is applied [25].

(1) $$Transmittance=\frac{Frame-Darkreferenceframe}{Whitereferenceframe-Darkreferenceframe}$$

**Step 2: Stitching**

Once the frames have been calibrated, each spectral band of each frame is arranged to form the raw hyperspectral cube. This arranging process is called stitching and the details of its operation are described in Section 2.2.3 of [23]. The final result of the stitching process is a raw hyperspectral cube with a resolution of 2048 × 1080 pixels with 192 virtual spectral bands, of which 145 will make up the final spectral cube.

**Step 3: Denoising and spectral correction**

The next step is mandatory according to the manufacturer because, due to the way the sensor is constructed, there are certain areas that do not capture useful spectral information. To convert from 192 virtual bands to 145 spectral bands, it is necessary to multiply the spectral cube by a correction matrix provided by the manufacturer. The hyperspectral cube’s resolution after this operation is 2048 × 1080 pixels, with the final 145 spectral bands.

**Step 4: Shifting correction**

The main challenge with wedge line scan cameras is that any inaccuracy in the capture process caused by the tolerance of the microscope’s linear actuators can result in a spatial shift in the hyperspectral cube. This means that, spatially, the first band of the cube may not coincide with the last one due to small shifts along the cube. To solve this, we apply, in turn, the following operations: first, calculate the shift between each band of the cube, then move each band to the correct position, and finally crop the padding added in each movement, which corresponds to the maximum calculated shift. If a shift threshold of 100 pixels is exceeded, the cube is discarded and must be recaptured. The shift is calculated using the OpenCV [26] function *phaseCorrelate*, and the movement is performed using *warpAffine*, also by OpenCV. This process reduces spatial resolution but ensures that the captured and preprocessed data are correct.

**Step 5: Normalization**

The final step is the normalization of the hyperspectral data by applying a min–max normalization as in Equation (2):

(2) $$X_{normalized}=\frac{value-min}{max-min}$$

**Step 6: RGB synthesis**

Once the hyperspectral cube is conformed, all of the spatial information of the samples is captured, with the spectral bands already aligned along the wavelength dimension. This makes it possible to generate the synthetic RGB image. Given that the range of the hyperspectral camera used in this work is between 470 and 890 nm, i.e., it captures bands in the visible range of the spectrum, it can synthesize an RGB image from the bands closest to the reference bands of the colors red, green, and blue. To achieve this, the selected bands are 462.14 nm for blue, 543.65 nm for green, and 605.61 nm for red.

### 2.2. Database Generation Methodology

**Figure 1:**
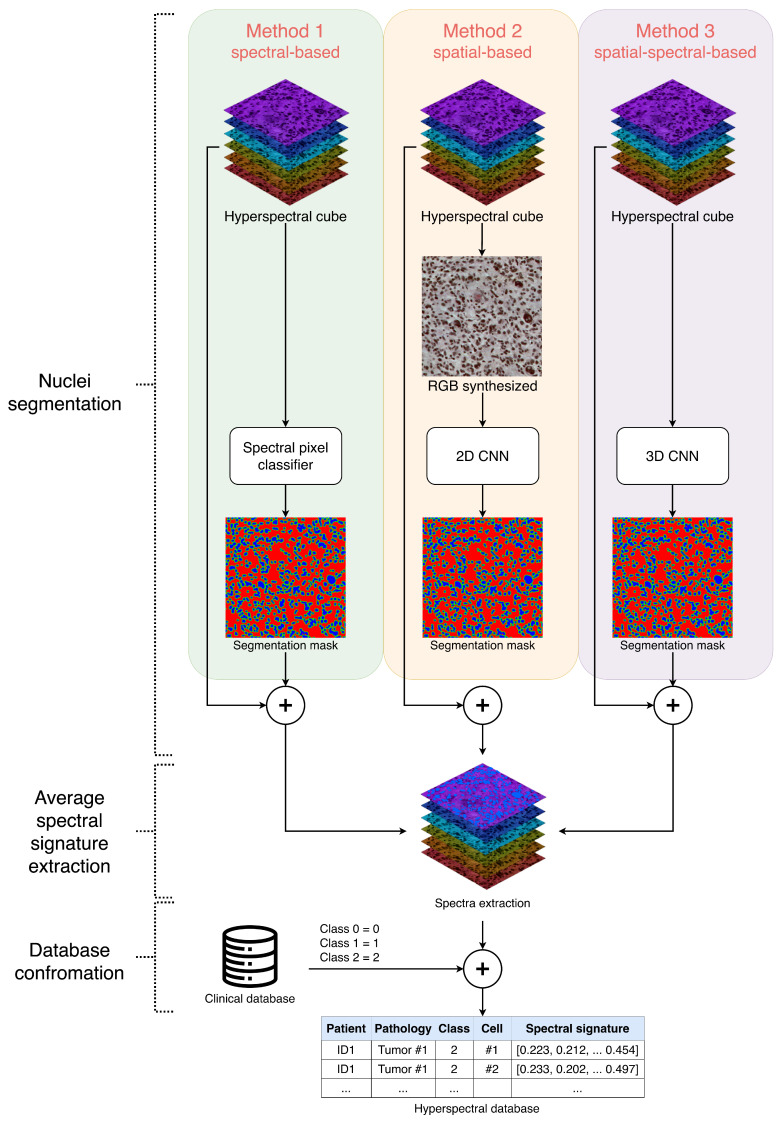
General diagram of the proposed methodology for generating hyperspectral databases based on cell nuclei segmentation. The three proposed segmentation methods are presented, based on spectral information (Method 1), spatial information (Method 2), and spatial–spectral information (Method 3).

In Figure 1, a general diagram of the proposed workflow for generating the hyperspectral database with the three proposed nuclei segmentation methods is presented. The objective of this method is to segment cell nuclei in order to generate a final segmentation map that will be overlaid onto the hyperspectral cube, enabling the extraction of spectral signatures from pixels belonging to the nuclei class. The average spectral signature is then computed for each cell, as extracting a single average signature per cell reduces the noise caused by random pixel-level variations within the same nucleus, yielding a more stable and representative spectral profile. Moreover, by avoiding the inclusion of multiple nearly identical pixel-level signatures, this approach reduces dataset dimensionality while increasing variance, thereby improving overall data quality. Thanks to this approach, each of these average spectral signatures can be auto-labeled based on the diagnosis associated with the patient stored in a clinical database. For example, for each average signature of a patient diagnosed with a certain type of tumor, all average spectral signatures for that sample will be labeled and supervised by expert pathologists according to the World Health Organization (2021) criteria [27].

#### 2.2.1. Nuclei Segmentation Method 1: Spectral-Only Pixel Classification

This method segments cell nuclei based on the spectral information of each of the pixels that make up the hyperspectral cube based on the machine learning algorithm XGBoost [28]. To perform this segmentation, the hyperspectral cube is flattened at the input so that the pixels become a two-dimensional array. Subsequently, the trained algorithm predicts each of the pixels to then generate the segmentation map with the spatial dimensions of the input hyperspectral cube.

To generate the ground truth masks needed for training, nuclei pixels from 30 hyperspectral cubes described in Section 2.1 were manually annotated. The annotation process was performed by three expert pathologists from Hospital Universitario 12 de Octubre (Madrid, Spain) to minimize inter-operator variability. Annotations were performed using the QuPath tool [29] (version 5.0.0) on synthetic RGB images.

The training and test splits were defined considering the intrinsic imbalance of the tissue samples, as tumor images typically contain a substantially higher number of cell nuclei than healthy ones. Consequently, the training set comprised 20 cubes (5 tumor and 15 healthy), while the test set consisted of 10 cubes equally distributed between tumor and healthy samples (5 tumor and 5 healthy) to ensure a fair evaluation. The number of extracted spectral signatures per split and pathology is summarized in Table 1, which reports the signatures before balancing and after undersampling-based balancing.

To maximize training performance, hyperparameter optimization was carried out using the Optuna framework [30], taking the region-based metric Dice Similarity Coefficient (DSC) of the nuclei class as the target metric. The hyperparameter ranges used are described in Table 2. The machine for training is a computational server with an Nvidia A100 80GB, 2× AMD EPYC 7317 16-Core Processor with 64 threads, and 1TB of RAM.

#### 2.2.2. Nuclei Segmentation Method 2: Spatial CNN-Based Segmentation from Synthetic RGB

This method performs cell nuclei segmentation using only the spatial information of the image, following an approach analogous to standard RGB image analysis. In this approach, only the synthetic RGB images of the dataset were used as inputs. We implemented a solution based on ConvNeXt-XL [31], a modern Convolutional Neural Network (CNN), the structure of which is based on a Visual Transformer (ViT) [32] architectures, and which has demonstrated strong performance for this task.

We use the ConvNeXt-XL implementation provided by the MMSegmentation framework [33], pretrained on the ImageNet-21k dataset [34]. Starting from this pretrained baseline, the network is fine-tuned using the same ground truth masks described in Section 2.2.1. In this case, however, the ground truth masks are modified to follow the methodology proposed by Salvi et al. [35], in which an additional auxiliary class corresponding to cell borders is introduced. This class enables the network to learn and generate cell boundaries, providing better separation between cell nuclei to avoid detecting clusters of nuclei as a single entity [36]. This is especially important in tumor tissue, as the intracellular distance and cell number increase much more than in non-pathological tissue. This border class is generated by applying a 3-pixel dilation to the contour of each nucleus. Consequently, the dataset used for this method comprises three classes: 0 for background, 1 for border, and 2 for nuclei.

For the fine-tuning training process, each synthetic RGB image along with their ground truth segmentation masks is cropped in patches of 640 × 640 pixels to match the size requirements of the base dataset used by the network. Cropping was performed without overlap. A strict no-leakage policy was enforced to ensure that all patches from an image assigned to the training set did not leak into the validation or test sets. Table 3 summarizes the division between the different splits at the patient and patch levels.

During training, hyperparameter optimization was carried out using the search ranges detailed in Table 4 to maximize network performance. The loss function used was cross-entropy weighted [37]. To increase data variability to improve the performance during training, a series of data augmentation techniques were applied: random rotations between 45 and −45 degrees, horizontal flips, vertical flips (both with a probability of 50%), and random brightness contrast (20% of probability). Training was carried out on the same server described in Method 1 in Section 2.2.1.

#### 2.2.3. Nuclei Segmentation Method 3: Spatial–Spectral 3D CNN Segmentation

While Method 1 performs nucleus segmentation based exclusively on spectral signatures extracted from the hyperspectral cube, and Method 2 exploits spatial information derived from synthetic RGB representations, Method 3 aims to jointly leverage both spatial and spectral information in a unified method. To this end, a three-dimensional U-Net (3D U-Net) architecture was implemented [38], where the hyperspectral data cube is treated as a 3D input, with the two spatial dimensions and the spectral dimension jointly processed by 3D convolutional kernels. This design enables the network to learn hierarchical features that capture spatial morphology while simultaneously modeling spectral correlations across adjacent wavelengths (across the entire range captured by our system), thus providing a principled way to exploit the full spatio-spectral structure of hyperspectral images.

The 3D U-Net architecture offers several advantages for this task. Its encoder–decoder structure with skip connections allows for the preservation of fine-grained spatial details while progressively incorporating higher-level contextual information, which is particularly relevant for delineating cell nuclei with heterogeneous shapes.

In order to obtain a single two-dimensional nuclei segmentation map from the volumetric network output, a modification was introduced in the final layer of the architecture. Specifically, an *AdaptiveAvgPool3d* (https://docs.pytorch.org/docs/stable/generated/torch.nn.AdaptiveAvgPool3d.html accesed on 9 January 2026) operation was applied along the spectral dimension, collapsing the spectral axis while preserving the spatial resolution of the feature maps. This operation aggregates spectral responses in a data-driven manner and enables the network to produce a final 2D segmentation mask consistent with the ground truth annotations, while still benefiting from three-dimensional feature extraction during the encoding and decoding stages. Furthermore, thanks to this modification, ground truth masks remain 2D masks, so no changes need to be made to them.

To train this network, the same dataset was used as in the previous method in Section 2.2.2, again with the classes: 0 for background, 1 for borders, and 2 for nuclei. Due to memory limitations of the GPU used for training, the hyperspectral cube crops were 64 × 64. Given that these crops were so small, a 25% overlap was used in patchify to expand spatial information. As in the previous method, a strict no-leakage policy was enforced to ensure that no patches from an image assigned to the training set could leak into the validation or test sets. All information about the experiment is summarized in Table 5.

Table 6 summarizes the hyperparameter search range used for the optimization of this network. The loss function was cross-entropy weighted. Training was carried out on the same server described in Method 2 in Section 2.2.2.

## 3. Results

To objectively analyze the three proposed cell nuclei segmentation methods, in this section we evaluate their performance quantitatively by analyzing their metrics on the test sets and the cell nuclei count, and also qualitatively by analyzing the segmentation maps generated.

### 3.1. Quantitative Cell Nuclei Segmentation Performance

To perform a quantitative evaluation of the three proposed methods, the test set of the dataset was predicted with the best trained model of each of the methods based on the DSC value for the nuclei class in the validation set. The pixel-based metrics chosen are precision, recall and F1 score, and the region-based metric is DSC. The results are shown in Table 7. It is important to mention that in both Method 2 and Method 3 metrics, the macro average was calculated only for the nuclei and background classes, since the border class is an auxiliary class that helps the network learn the boundaries of the cell nuclei.

As can be observed in Table 7, all approaches achieve high performance for the background class, while lower scores are observed for nuclei, reflecting the intrinsic difficulty of accurate nucleus delineation. The spectral-only method performs competitive pixel-wise metrics despite ignoring spatial context, whereas the spatial and spatial–spectral approaches yield comparable F1 and DSC scores for the nuclei class. Overall, pixel-level metrics alone suggest relatively similar segmentation performance across methods.

### 3.2. Cell Count Analysis

To assess whether the different segmentation strategies correctly preserve individual cell instances, a cell count analysis was performed by comparing the number of detected nuclei with the ground truth annotations for each test image. In this way, we can also obtain a metric that evaluates a more biological context of the dataset. The results are reported in Table 8. To perform the cell count, a contour analysis using the function *findContour* from OpenCV was performed for each of the segmentation maps generated on the test set, using only the pixels belonging to the nuclei class. By analyzing the contours of only nuclei pixels, the number of perfectly closed contours was counted and compared with the number of nuclei annotated by expert pathologists in the ground truth masks, computing the variance for each test image.

Table 8 shows that the purely spectral method (Method 1) causes massive overdetection of nuclei cells, especially in healthy tissue images (images 4, 5, and 6) since pixel-wise classification is very noisy. The spatial method (Method 2) shows much better performance than Method 1, but indicates its underdetection with a mean variance of around 30% with respect the ground truth in tumor tissue images, which has a higher cell density per µm^2^. Method 3 demonstrates the best detection of the three solutions in both healthy and tumor tissue images, with a mean variance from the ground truth of −4.25%.

### 3.3. Qualitative Comparison of Segmentation Results

To complete the analysis of results, Table 9 presents a series of random selected 640 × 640 crops from each test images along with their ground truth mask and the segmentation maps generated from the best model of each of the three methods. The synthesized RGB image has also been added for better comparison by the reader.

The qualitative analysis that can be extracted from crops in Table 9 indicates that the method based purely on the spectral part (Method 1) is excessively noisy. It can be observed how, around the detected cells, there are pixels incorrectly predicted as nuclei. Furthermore, in the images of tumor tissue (1, 2, and 3), it can be observed how cell detection tends to agglomerate, forming large cell clusters instead of segmenting them individually. Method 2 (purely spatial) and Method 3 (spatial-spectral) significantly correct this noise and also generate more coherent and morphologically consistent cell nuclei. However, it can be observed how Method 2 still tends to form nuclei clusters in the nuclei that are much more closely together in tumor tissue images (with higher nuclear density per µm^2^).

## 4. Discussion

Hyperspectral imaging is a powerful technology for clinical histology, as it enables spatial analysis comparable to conventional RGB imaging while substantially extending the available information by capturing a certain range of the electromagnetic spectrum. To perform reliable spectral analysis of pathological samples, it is essential to generate databases of spectral signatures specifically associated with cell nuclei, since the background in histological images is typically highly heterogeneous and spectrally noisy. Nuclear morphology is also key in the diagnosis of tumor cells and can enable standardization of diagnosis. It also tends to show small changes that are not observable to the pathologist’s eye, as they are associated with structural loss, changes in DNA quantity, or even chromatin condensation. For all these reasons, this work presents a comprehensive methodology for constructing hyperspectral databases of cell nuclei from histopathological samples.

The primary objective of the proposed methodology is to accurately extract meaningful spectral information from raw hyperspectral data cubes. To this end, Section 2.1.2 and Section 2.1.3 describe the hyperspectral acquisition system and its validated preprocessing pipeline. Once the hyperspectral cubes are obtained, the critical step becomes the accurate segmentation of cell nuclei. Accordingly, three segmentation strategies are investigated: a spectral-only approach that relies exclusively on pixel-wise spectral information (Section 2.2.1), a spatial-only approach based on features extracted from synthetic RGB images derived from the hyperspectral cubes (Section 2.2.2), and a spatial–spectral approach that jointly exploits spatial and spectral information (Section 2.2.3). Regardless of the segmentation method chosen, each one produces produces a nuclei segmentation map, from which individual cells are identified by grouping spatially adjacent nuclear pixels. In the final step of the proposed pipeline, the average spectral signature of each segmented nucleus is computed and automatically labeled using the available clinical information, resulting in a structured and clinically meaningful hyperspectral database.

To evaluate the proposed segmentation strategies, a proprietary hyperspectral dataset of brain histopathological samples comprising both tumor and healthy tissue was constructed. The dataset includes 30 hyperspectral image cubes (10 tumor and 20 healthy), which were split into 20 cubes for training, four for validation, and six for testing (see Table 3). Three expert pathologists independently annotated the dataset, providing the ground truth segmentation masks. Each method was trained by optimizing the DSC for the nuclei class, and performance was evaluated on the test set. Quantitative segmentation metrics and qualitative visual results are reported in Section 3.1 and Section 3.3, respectively. In addition, an instance-level analysis was conducted by comparing the number of segmented nuclei with the ground truth cell counts, as presented in Section 3.2.

Together, these results provide a comprehensive characterization of the strengths and limitations of each segmentation approach. The spectral-only method (Method 1) exhibits highly noisy behavior across both healthy and tumor tissues, with particularly extreme deviations in healthy samples. As shown in Table 8, the number of detected nuclei in healthy cubes exceeds the ground truth by several orders of magnitude (up to 7702%), indicating severe fragmentation of homogeneous nuclear regions into multiple small disconnected components. This behavior is consistent with the qualitative segmentation maps shown in Table 9, where it can be observed how there is a very significant number of pixels marked as background that are incorrectly predicted as core by the algorithm, especially in the healthy cubes (4, 5, and 6). Despite being the only method optimized using the Optuna framework to maximize the DSC of the nucleus class in the validation set, this approach yields the poorest overall performance. In particular, it achieves a DSC of only 61.89% for the nucleus class (Table 7). For this method to be viable in a production setting, a robust post-processing pipeline would be required, primarily focused on aggressive denoising of the predicted segmentation maps. Even with such corrections, the limited ability of the method to accurately separate individual nuclei would likely remain a critical bottleneck.

In contrast, the spatial-only (Method 2) and spatial–spectral (Method 3) approaches benefit from explicitly modeling the spatial context. Both methods incorporate an auxiliary nuclei border class, which enables the neural networks to learn the physical contours of nuclei and improves their separation during the spectral signature extraction stage. Method 2 achieves the highest pixel-wise segmentation performance, with DSC values of 78.97% for the nucleus class and 94.32% for the background. However, the cell count analysis reveals a substantial limitation: this method underestimates the number of nuclei by approximately 30% in tumor tissue, which typically exhibits a higher cell density than healthy tissue. Inspection of the corresponding segmentation maps indicates that this behavior arises from the tendency of the model to merge adjacent nuclei, thereby reducing its reliability for instance-level analysis and morphological studies. By comparison, Method 3 achieves slightly lower pixel-wise performance, with DSC values of 73.13% for nuclei and 93.16% for background, but demonstrates significantly greater robustness in correctly separating individual cell nuclei. Its average deviation from the ground truth cell count is approximately 4%, indicating a much closer correspondence with expert annotations. This improved instance-level accuracy is further corroborated by qualitative comparisons, where the segmentation maps produced by Method 3 closely resemble the ground truth delineations and avoid the large nuclear clusters observed with Method 2.

From a broader biomedical imaging perspective, these findings reinforce the principle that performance metrics must be interpreted in the context of their downstream analytical implications. Systematic reviews in quantitative biomedical imaging have emphasized that preprocessing and segmentation stages fundamentally shape the robustness, reproducibility, and translational value of derived biomarkers and structured databases [39,40]. Furthermore, Ghai et al. [41] reinforces the idea that the use of standardized metrics not only improves reproducibility but also has the potential to optimize patient care by facilitating more accurate comparisons between different domains and populations in medical research. Inadequate instance-level delineation may introduce systematic bias in feature extraction, compromise statistical validity, and limit cross-cohort comparability. Therefore, segmentation strategies should be evaluated not only according to conventional pixel-wise metrics but also based on their capacity to preserve biologically and clinically meaningful structures, particularly when the ultimate objective is database construction or biomarker development.

## 5. Conclusions and Future Work

Although hyperspectral imaging provides rich spectral information, accurate nucleus segmentation is primarily driven by spatial morphology. Spectral information alone is insufficient for robust instance delineation and, when naively combined with spatial features, does not necessarily yield improved performance in data-limited scenarios. The results presented in this work highlight that pixel-wise segmentation accuracy does not necessarily translate into biologically meaningful instance-level separation, underscoring a broader methodological consideration in biomedical image analysis—isolated performance metrics may fail to capture functional validity when downstream quantitative interpretation is required.

Based on these findings, the nuclei segmentation based on spatial–spectral information offers the most reliable and practical solution for nuclei delineation and spectral signature extraction. Future work will focus on improving the performance of this method and be tested on a clinical study-scale database. Furthermore, in the medium term, the objective will be to test this method on other tissues and with other staining protocols in order to standardize this method within a much more general framework.

## Figures and Tables

**Table 1:** Summary table of the total number of spectral signatures from the training cubes before undersampling versus the number of balanced signatures used during training.

	Class	Spectral Signatures
Before undersampling	Background	35,643,517 (85.11%)
	Nuclei	6,234,563 (14.89%)
	Total	41,878,080 (100%)
After undersampling	Background	6,234,563 (50%)
	Nuclei	6,234,563 (50%)
	Total	12,469,126 (100%)

**Table 2:** Search range of hyperparameters used by Optuna to optimize XGBoost.

Hyperparameter	Range/Values	Final Value
Number estimators	[100:2000]	1187
Max depth	[5:15]	5
Learning rate	[0.01:0.5]	0.011

**Table 3:** Summary table of the training, validation, and test split of the dataset for fine-tuning the ConvNeXt-XL CNN for nuclei cell segmentation.

Dataset	Class	Number of Patients	Number of 640 × 640 Crops
Training set	Tumor	5	40
	Healthy	15	112
	**TOTAL**	**20**	**152 (65%)**
Validation set	Tumor	2	16
	Healthy	2	16
	**TOTAL**	**4**	**32 (14%)**
Test set	Tumor	3	24
	Healthy	3	24
	**TOTAL**	**6**	**48 (21%)**

**Table 4:** Search ranges of the hyperparameters applied to the ConvNeXt-XL network in method 3 during fine-tuning training process.

Hyperparameter	Range/Values	Final Value
Learning rate	$\left[x\times 10^{-i}|x\in \left[1:9\right],\;i\in \left[2:6\right]\right]$	$1\times 10^{-2}$
Batch size	[4, 8, 16, 24, 32]	24
Epochs	[5:200]	50

**Table 5:** Summary table of the training, validation, and test split of the dataset for fine-tuning the 3D U-Net CNN for nuclei cell segmentation.

Dataset	Class	Number of Patients	Number of 64 × 64 Crops (25% Overlap)
Training set	Tumor	5	4305
	Healthy	15	12,915
	**TOTAL**	**20**	**17,220 (67%)**
Validation set	Tumor	2	1722
	Healthy	2	1722
	**TOTAL**	**4**	**3444 (13%)**
Test set	Tumor	3	2583
	Healthy	3	2583
	**TOTAL**	**6**	**5166 (20%)**

**Table 6:** Search ranges of the hyperparameters applied to the 3D U-Net network in method 3.

Hyperparameter	Range/Values	Final Value
Learning rate	$\left[x\times 10^{-i}|x\in \left[1:9\right],\;i\in \left[1:4\right]\right]$	$3\times 10^{-2}$
Batch size	[2, 4, 6, 8, 10]	10
Epochs	[5:100]	25

**Table 7:** Summary table of pixel-wise metrics for each of the cell nucleus segmentation methods in the test set.

Method	Classes	Pixel-Based Metrics			Region-Based Metrics
		Precision	Recall	F1 Score	DSC
**1**	Background	93.00%	82.23%	87.28%	84.42%
	Nuclei	56.16%	78.61%	65.52%	61.89%
	**Average**	74.58%	80.42%	76.40%	73.16%
**2**	Background	96.77%	91.99%	94.32%	94.32%
	Border	51.50%	68.95%	58.96%	58.96%
	Nuclei	79.48%	78.47%	78.97%	78.97%
	Average *	88.12%	85.23%	86.64%	86.64%
**3**	Background	96.44%	93.44%	94.92%	93.16%
	Border	54.86%	68.62%	60.98%	55.98%
	Nuclei	79.70%	76.43%	78.03%	73.13%
	Average *	77.00%	79.50%	77.97%	74.09%

* Average computed only on main classes: nuclei and background.

**Table 8:** Number of cells detected by each nuclei segmentation method in the test set images. From left to right, the image number of the test set, the number of cells in the ground truth mask (GT cells), and the number of cells detected by each method are shown, along with their variance with respect to the ground truth (Δ). The test images belong to tumor tissue (Tu) and healthy tissue (He).

Test Cube	GT	Method 1		Method 2		Method 3	
	Cells	Cells	Δ	Cells	Δ	Cells	Δ
Cube 1 (Tu)	2769	1358	−50.96%	1980	−28.49%	2809	1.44%
Cube 2 (Tu)	2304	6666	189.32%	1534	−33.42%	2142	−7.03%
Cube 3 (Tu)	3119	9691	210.71%	2127	−31.81%	2579	−17.31%
Cube 4 (He)	577	45,018	7702.08%	529	−8.32%	538	−6.76%
Cube 5 (He)	622	13,172	2017.68%	594	−4.50%	616	−0.96%
Cube 6 (He)	352	13,334	3688.07%	338	−3.98%	370	5.11%
Average			2292.82%		−18.42%		−4.25%

**Table 9:** Qualitative results of the proposed methods on the test set hyperspectral cubes, together with the synthesized RGB image and the ground truth mask annotated by expert pathologists. The images are 640 × 640 crops. Red pixels belongs to the background class, green pixels to the border class (only in methods 2 and 3), and blue pixels belongs to the nuclei class. The test images belong to tumor tissue (Tu) and healthy tissue (He).

Cube	RGB Synthesized	GT Mask	Method 1	Method 2	Method 3
Cube 1 (Tu)	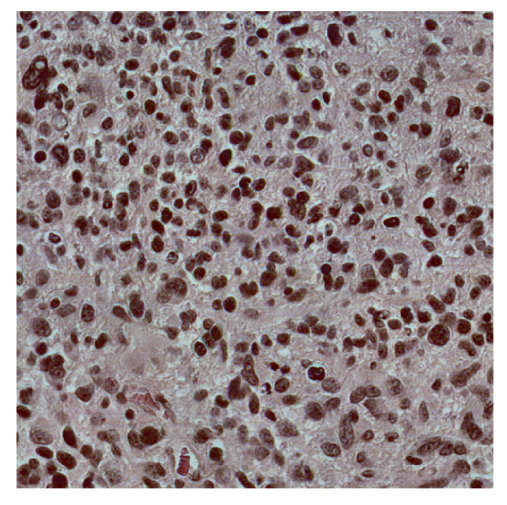	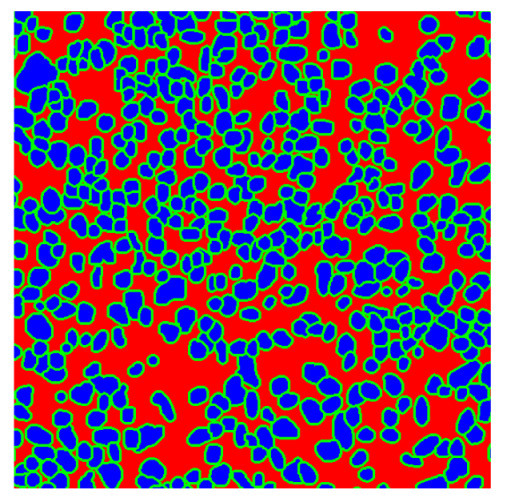	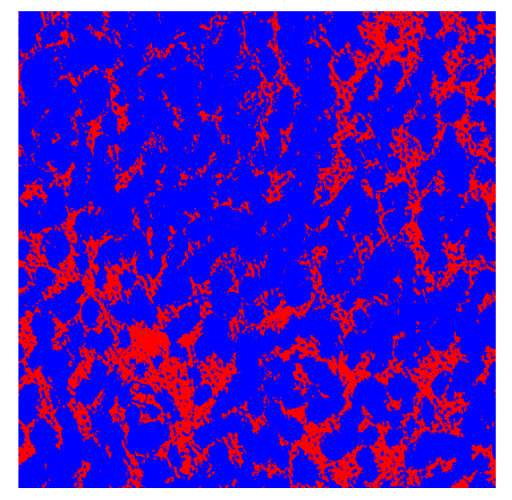	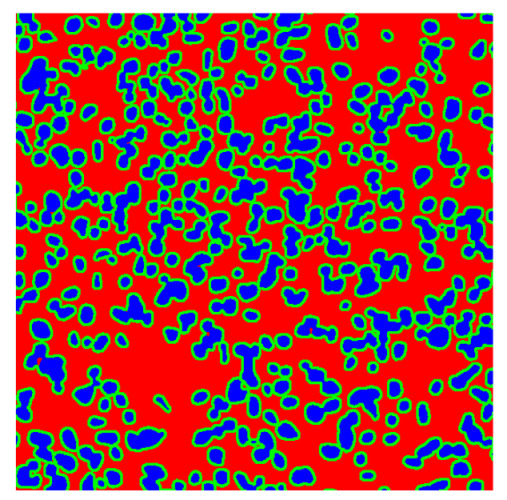	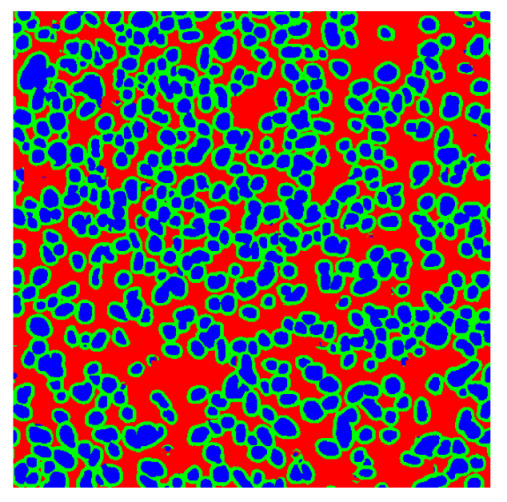
Cube 2 (Tu)	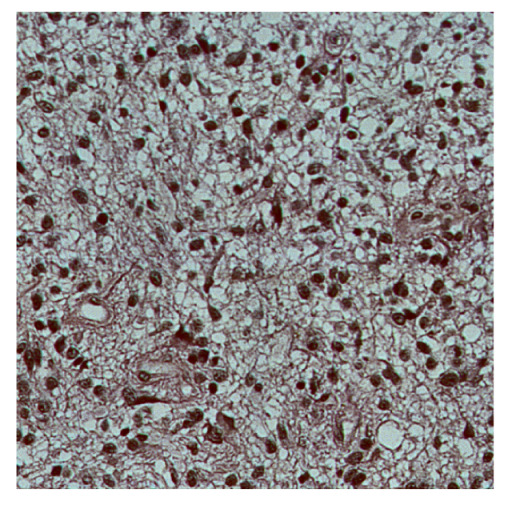	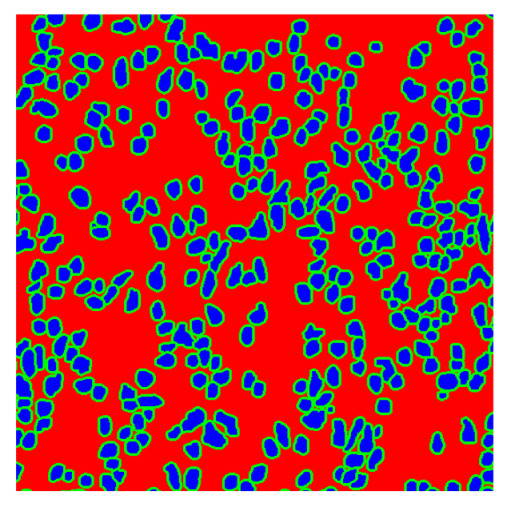	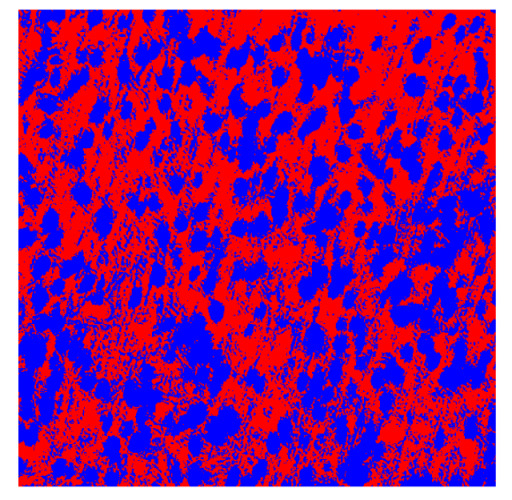	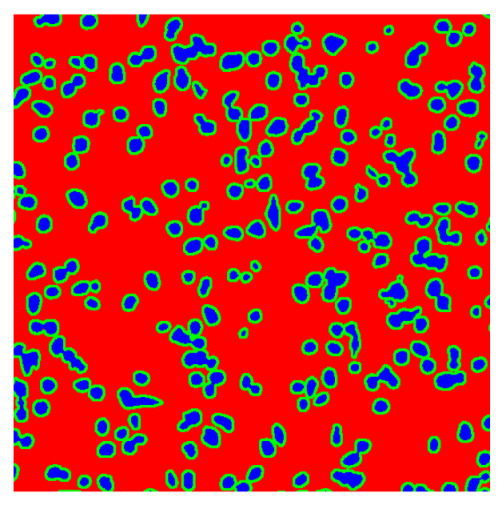	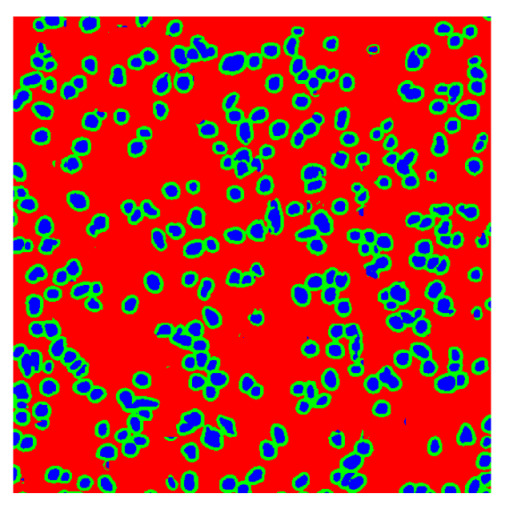
Cube 3 (Tu)	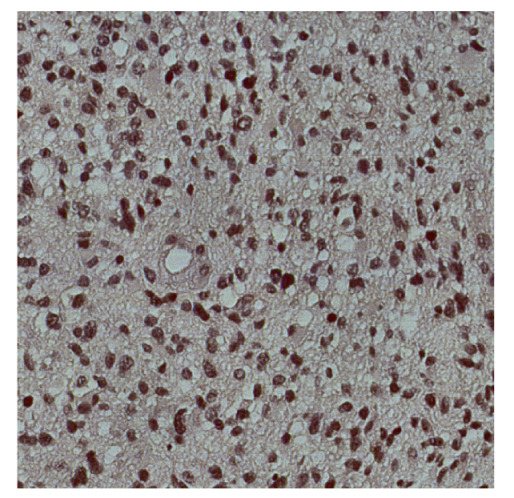	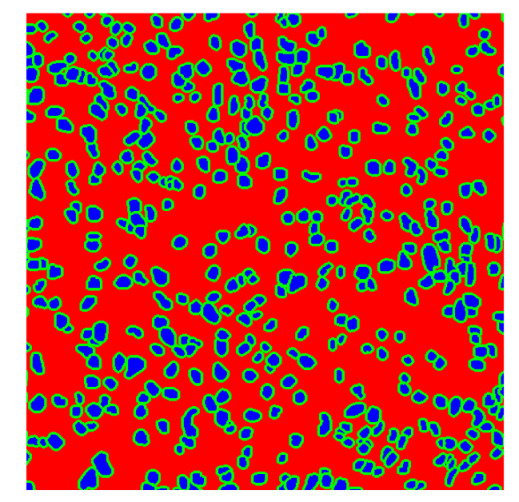	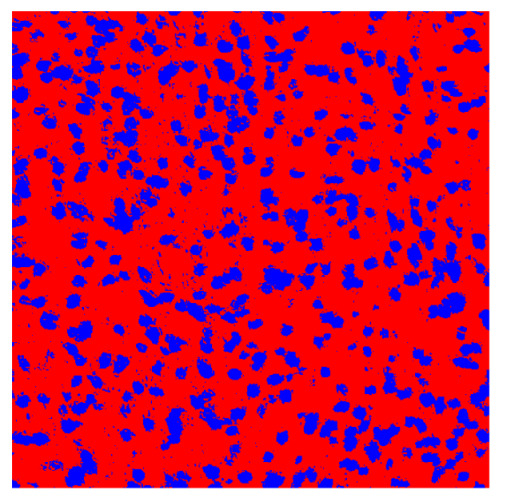	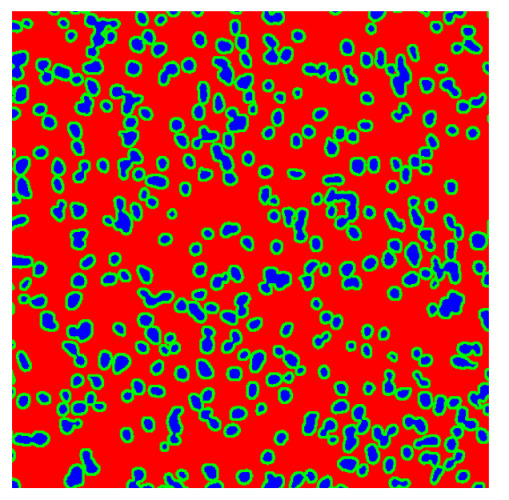	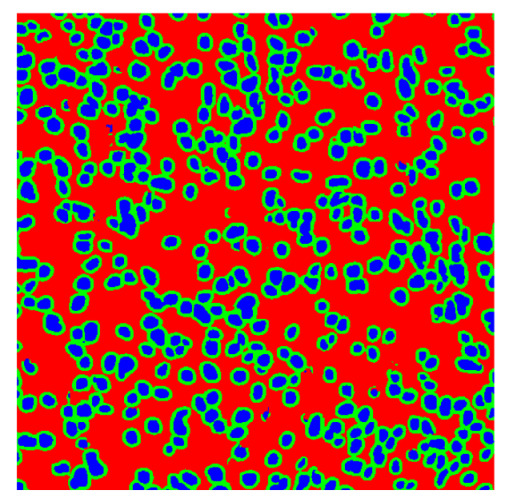
Cube 4 (He)	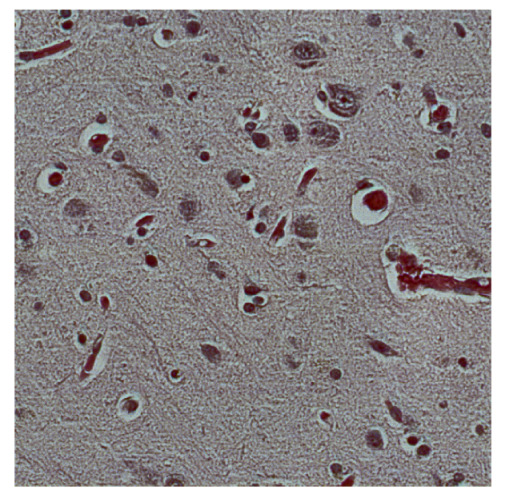	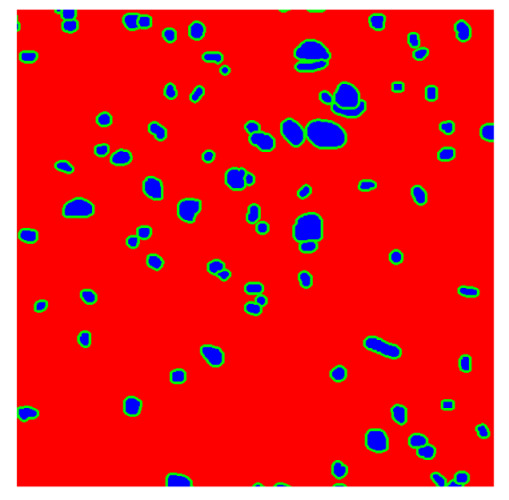	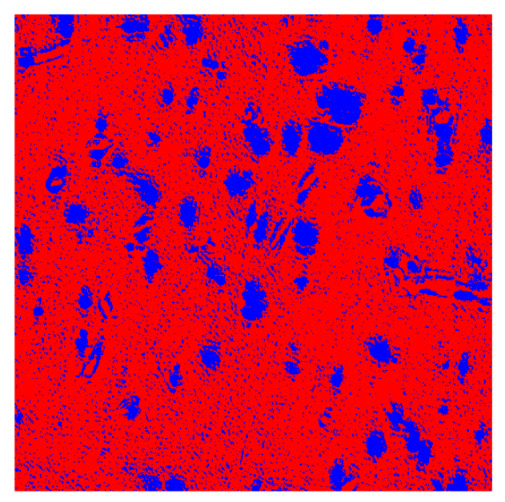	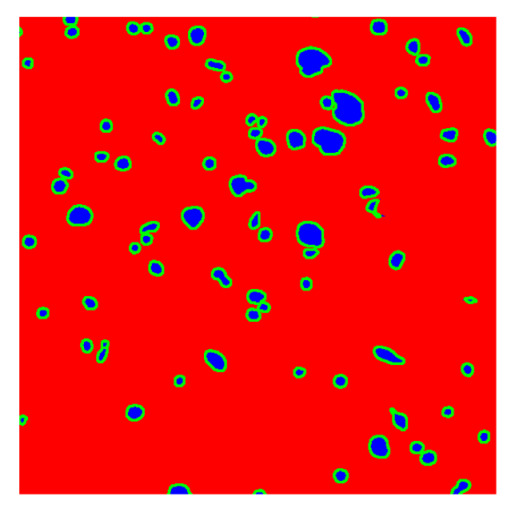	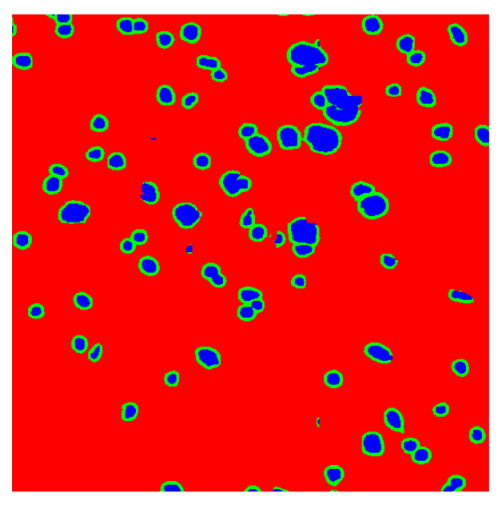
Cube 5 (He)	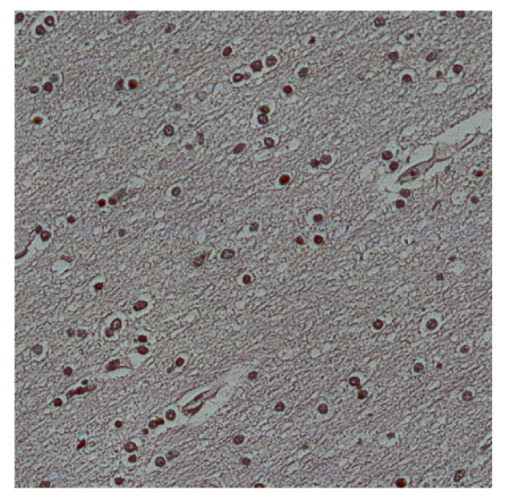	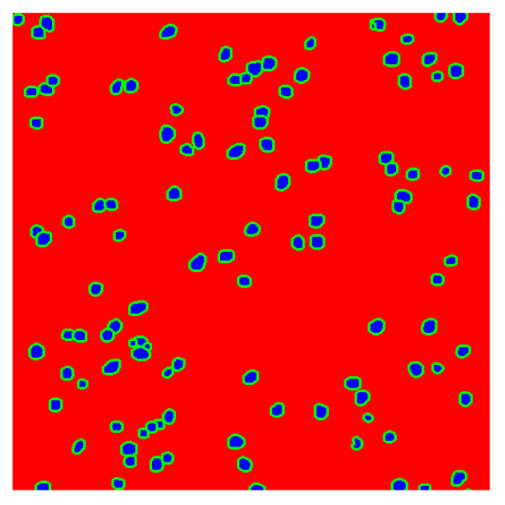	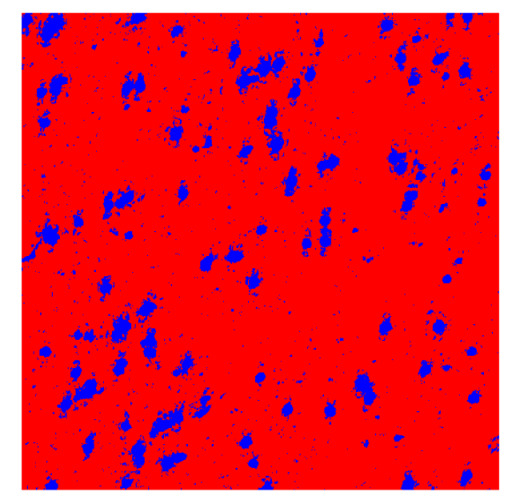	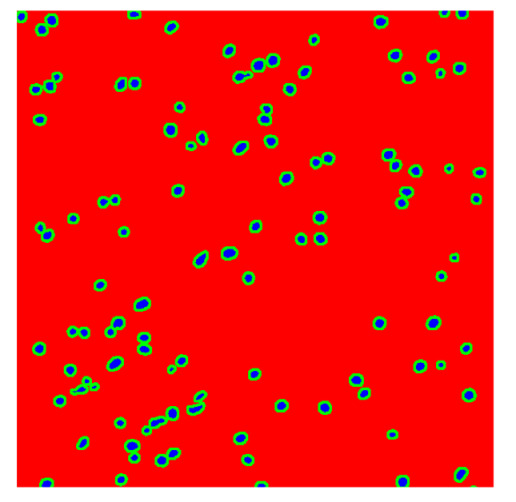	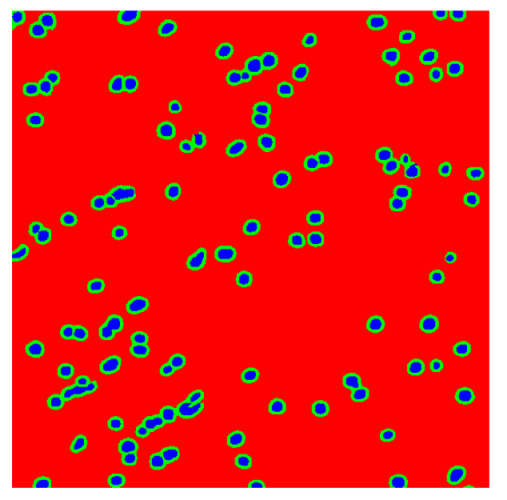
Cube 6 (He)	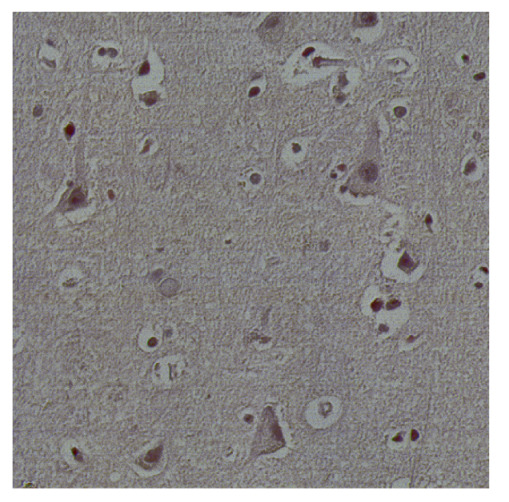	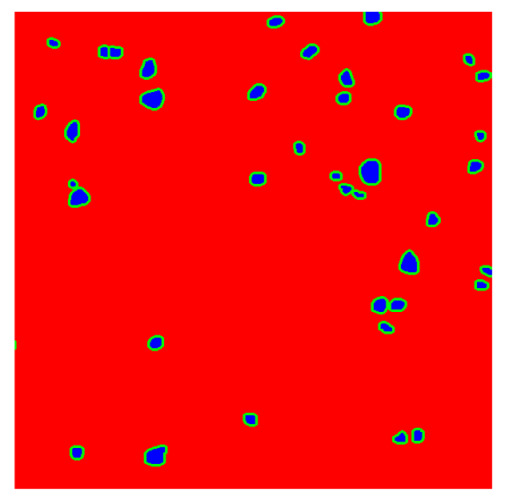	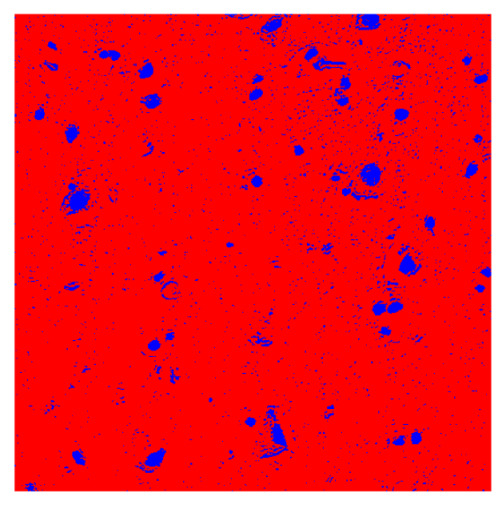	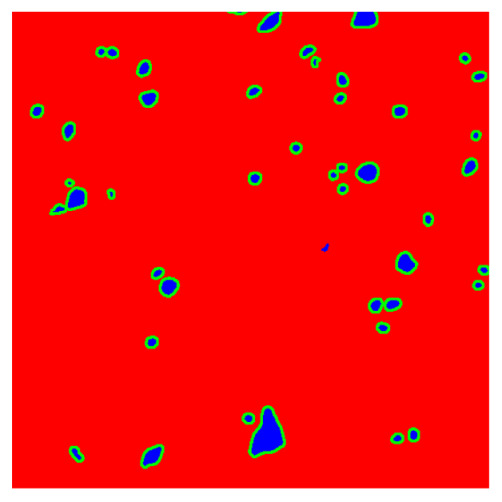	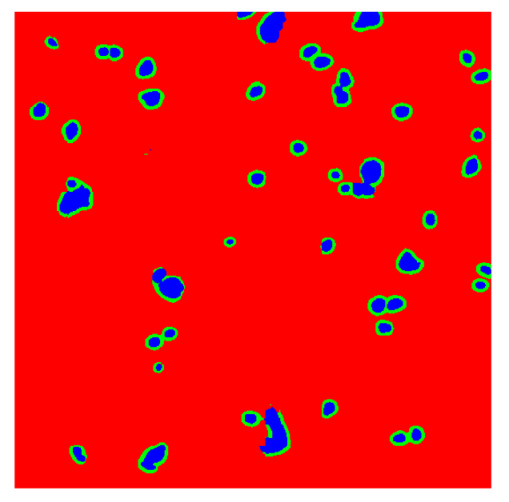

## Data Availability

The data cannot be made publicly available upon publication due to legal restrictions preventing unrestricted public distribution. The data that support the findings of this study are available upon reasonable request to the authors.

## Abbreviations

The following abbreviations are used in this manuscript:

DL	Deep Learning
HSI	Hyperspectral imaging
WSI	Whole-slide imaging
RGB	Red, Green, and Blue
DSC	Dice Similarity Coefficient
CNN	Convolutional Neural Network
H&E	Hematoxylin and Eosin
